# Advances in Analysis and Detection of Major Mycotoxins in Foods

**DOI:** 10.3390/foods9040518

**Published:** 2020-04-20

**Authors:** Sofia Agriopoulou, Eygenia Stamatelopoulou, Theodoros Varzakas

**Affiliations:** Department of Food Science and Technology, University of the Peloponnese, Antikalamos, 24100 Kalamata, Greece; sagriopoulou@gmail.com (S.A.); estamatel@gmail.com (E.S.)

**Keywords:** mycotoxins, analysis, detection, biosensors, aptamer, LC–MS/MS, sample preparation, hyperspectral imaging, electronic nose, quantitative NMR

## Abstract

Mycotoxins are the most widely studied biological toxins, which contaminate foods at very low concentrations. This review describes the emerging extraction techniques and the current and alternatives analytical techniques and methods that have been used to successfully detect and identify important mycotoxins. Some of them have proven to be particularly effective in not only the detection of mycotoxins, but also in detecting mycotoxin-producing fungi. Chromatographic techniques such as high-performance liquid chromatography coupled with various detectors like fluorescence, diode array, UV, liquid chromatography coupled with mass spectrometry, and liquid chromatography-tandem mass spectrometry, have been powerful tools for analyzing and detecting major mycotoxins. Recent progress of the development of rapid immunoaffinity-based detection techniques such as immunoassays and biosensors, as well as emerging technologies like proteomic and genomic methods, molecular techniques, electronic nose, aggregation-induced emission dye, quantitative NMR and hyperspectral imaging for the detection of mycotoxins in foods, have also been presented.

## 1. Introduction

Mycotoxins are by-products of secondary metabolism of filamentous fungi that cause harmful effects on human and animal health resulting in significant economic losses [1]. Important mycotoxins are aflatoxins (AFs), ochratoxin A (OTA), zearalenone (ZEN), fumonisins (FBs), ergot alkaloids (EAs), enniatins (ENs), patulin (PAT), *Alternaria* toxins (ATs) and trichothecenes (TCs) such as deoxynivalenol (DON), T-2 and HT-2 toxins (T-2, HT-2) [2,3]. The main producers of mycotoxins are the fungi of the genera of *Aspergillus, Fusarium, Penicillium, Claviceps* and *Alternaria* [4]. The appearance of toxigenic fungi and the subsequent production of mycotoxins are more frequently observed in food and feed produced in developing countries due to the climate, poor production practices and technologies and poor storage conditions for crops, but mycotoxin-contaminated food and feed can occur anywhere in the world through international trade [5]. Many agricultural products such as nuts [6], fresh and dried fruits and vegetables [7,8], cereals such as like maize, rice, and wheat [9], liquids such as wine, grape juice [10] and beer [11], milk and dairy products [12], spices and herbs [13], coffee and cocoa [14,15], and feed [16] can be contaminated with mycotoxins at all stages of the food and feed chain. Among mycotoxins with a wide range of toxic biological activities [1], aflatoxins, the most studied mycotoxins, exhibit carcinogenic, mutagenic, teratogenic and immunosuppressive effects [17], while aflatoxin AFB1 has been characterized as 1 carcinogen (carcinogenic to humans) according to the International Agency for Research on Cancer (IARC) [18].

Trustworthy and sensitive analysis of mycotoxins requires the application of an appropriate and certified procedure for detection and qualification, because mycotoxins can express their toxicity at low-dose levels. Regarding the isolation, separation and sample extraction procedure of mycotoxins, besides the traditional mycotoxin extraction methods with organic solvents, different means and methods have been used, such as Quick Easy Cheap Rough and Safe (QuEChERS), liquid–liquid extraction (LLE), solid–liquid extraction (SLE), accelerated solvent extraction (ASE), supercritical fluid extraction (SFE), microwave-assisted extraction (MAE), vortex assisted low density solvent–microextraction (VALDS–ME), solid phase extraction (SPE), BSA (bovine serum albumins)-based sample clean-up columns, aptamer-affinity columns (AACs), molecularly imprinted polymers (MIPs) and immunoaffinity columns (IACs) [5,19,20,21,22]. 

Many analytical techniques have been used from the very early discovery of mycotoxins till now, such as thin-layer chromatography (TLC), high-performance liquid chromatography (HPLC) in combination with different detectors (e.g., fluorescence, diode array, UV), liquid chromatography coupled with mass spectrometry (LC–MS), liquid chromatography-tandem mass spectrometry (LC–MS/MS) and gas chromatography-tandem mass spectrometry (GC–MS/MS) for mycotoxin analysis, with chromatographic techniques being dominant [19]. In most cases the extracted samples are analyzed by the LC–MS chromatographic method. In addition, the development of the LC–MS/MS technique for the simultaneous identification of multiple mycotoxins has achieved much attention in recent years [5,23].

On the other hand immunoassay-based methods, like enzyme-linked immunosorbent assay (ELISA) [22] and lateral-flow devices (LFDs) [24], are important methods when rapid analysis of mycotoxins is required. Also biosensors are a very useful tool for mycotoxins identification [25,26,27]. More emergent, recent and novel techniques for the detection and analysis of mycotoxins in foods can be performed by proteomic and genomic methods, molecular techniques, electronic nose [28,29] and hyperspectral imaging (HSI) [30,31].

In order to significantly reduce matrix effects, critical steps such as extraction, purification and chromatographic separation should be properly defined [32]. In the immunoassay-based methods, samples with color compounds that have not been properly pretreated could affect the sensitivity of detection of mycotoxins and overestimating results, as the matrix effects can interfere in the reading of results [33]. The analyte and the matrix determine the effect of the matrix, so the application of HPLC after immunoaffinity clean-up should be validated for each matrix/mycotoxin combination [34]. Moreover, the coelution of matrix components in LC–MS analysis suppresses or enhances the chromatographic signals [32]. 

The purpose of this review is to discuss the latest and innovative techniques applied in the analysis and determination of important mycotoxins in foods. Moreover, the most recent extraction methodologies along with clean-up procedures are presented.

## 2. Extraction Solutions, Extraction Methodologies and Clean-Up Procedures of Mycotoxins

At present, sample preparation focuses on finding environmentally friendly solvents, simplifying the process, and obtaining rapid results [20]. The most crucial steps before the mycotoxin analysis are the extraction method and clean-up. The extraction of the contaminated food and feed samples is intended to remove mycotoxins from the sample using appropriate solvents. The choice of solvents, as well as the method of extraction, contribute significantly to the success of the extraction. A suitable extraction solvent is one that removes only the mycotoxins from the sample with the highest efficiency. It is inexpensive, safe to the user, and reduces matrix effect. Mixtures of methanol–water and acetonitrile–water at different ratios, are the most frequently used extraction solvents in mycotoxin analysis [22]. Pigments, essential oils and fatty acids that are present in the samples make extraction difficult and require the use of different extraction solvents, such as a mixture of ethyl acetate–formic acid [35]. Moreover, other extraction solvents, like 1-octanol and toluene [20], dichloromethane [36], acetone [37] and chloroform [19] have been used to extract mycotoxins. Hydrophobic mycotoxins can be easily dissolved in all previous solvents, while polar mycotoxins, like FBs, are soluble in water [19]. Deep eutectic solvent (DES) was recently reported by He et al. [38] as a new green solvent, which successfully extracted aflatoxins from rice samples. This extractant was synthesized from the combination of two biodegradable, safe, and economical components, tetramethylammonium chloride and malonic acid. Moreover the use of this extract had limited the use of solvents and derivatization reagents which complies with the principles of green chemistry. 

Emerging extraction techniques with many advantages have been developed in mycotoxin analysis. The QuEChERS method was initially developed for pesticide analysis [39], but its use has also been reported for mycotoxin analysis, with high quality results [40,41,42]. This extraction technique uses small amounts of acetonitrile as an organic solvent, is economical, fast and does not require specialized personnel. In LLE, two immiscible phases are used from two different solvents, in which the mycotoxins exhibit different solubility. In one phase the nontarget substances are removed, and in the other phase, the mycotoxins are removed [43]. This method is usually common in liquid samples, while the SLE is used for mycotoxin analysis in agricultural products with solid structures, and mainly uses ultrasonic extraction, homogenization and shaking [22]. An ecofriendly, fast and efficient extraction technique is ASE, which uses traditional solvents and temperature above the boiling point. ASE is synonymous with pressurized liquid extraction (PLE), as it works in pressures at the range of 1500–2000 psi [37]. The properties of supercritical fluids are used in the technique of SFE, with the primary use of CO_2_ under critical conditions [37]. MAE has been characterized as a green technique, uses few organic solvents, and requires minimal extraction time. According to this technique, the organic solvents are heated rapidly by microwave energy and mycotoxins are separated into the solvent [44]. The VALDS–ME usually uses three different solvents, namely an extraction solvent, a dispersive solvent, and water. In a recent study by Somsubsin, et al., two nontoxic solvents, 1–octanol and toluene, were the extractant, and with the use of vortex and the addition of Na_2_SO_4_ as a demulsifier, very quick extraction of AFs from rice samples was achieved [20]. Table 1 summarizes all these extraction methods and solvents, along with the benefits and limitations of each one.

Before the analysis, the obligatory step of clean-up and sample concentration is followed. The clean-up step following extraction, helps to increase the selectivity as it contributes to the further removal of interference from the matrix [49]. The SPE technique is very popular and fast, requires less solvent, and purifies the mycotoxins by sorption on a solid absorbent. The solid absorbents, usually packed in cartridges, are rinsed with the purpose of removing contaminants and capturing the mycotoxin [15]. Except from the conventional strong anionic exchange cartridges and C-8, C−18, hydrophilic–lipophilic balance cartridges, novel and interesting absorbents have been developed for mycotoxin clean-up [22]. Carbon nanomaterial and magnetic carbon nanomaterial were recently used as alternative sorbent material with high-absorption capacities. Among them, graphene oxide was used for analysis of AFs [50], and multi-walled carbon nanotubes were used simultaneously for analysis of TCs [51]. The unique electronic, mechanical and chemical properties of carbon nanomaterials make them advantageous materials with high absorption capacity. Regarding the limitations of the new alternative sorbent materials over the old ones, the self-synthesis of these materials could limit their scope, while in order to be considered appropriate, they must be evaluated in various types of mycotoxins [22].

Clean-up is usually done through IACs in order to receive better limits of quantification, precision and accuracy. IACs have packed material with specific antibodies for certain mycotoxins, which interact with the antigens of the test sample when passing through the column. At the same time, the interfering compounds contained in the sample are removed by column washing, and the mycotoxin is eluted with a usually miscible solvent [5]. The IAC clean-up has been a widely used method, both for one mycotoxin and multimycotoxin analysis. For multimycotoxin analysis, multimycotoxin IACs which simultaneously analyze AFs, OTA, ZEA, and *Fusarium* toxins have been developed [52,53]. Although the use of IACs give trustworthy results, mycotoxin analysis with IACs takes long time, and many toxic organic reagents are used [54]. These columns may have a limited ability to absorb mycotoxins, either due to the numerous components contained in the matrix that interfere with the antibodies, or due to their inhibition against mycotoxin structures, preventing them from binding to the antibodies. Also, IACs have a short life, and they are expensive [55]. 

The BSA-based sample clean-up columns are also used for the clean-up of mycotoxins. Serum albumins, especially those of bovine origin, are cheap and widespread. They have strong affinity with some mycotoxins and can be used in the step of clean-up. A novel BSA-based sample clean-up column was developed and validated by Leal et al. [21] in order to be used to determine OTA in wine. The method was based on the satisfactory bind of OTA with BSA, after immobilization on agarose. The implementation of the method was satisfactory when compared to IACs. Serum albumin, a type of globular protein, forms strong complexes with OTA [56]. OTA interference due to its binding to the protein may reduce or enhance the assays results, and this is a major disadvantage of using BSA as a blocking agent [21].

Synthetic systems have been developed, such as MIPs, aptamers and peptides to replace the antibodies in IACs [15,57,58]. MIPs are synthetic polymers highly specialized in identifying selective analytes, and are cheaper than antibodies [22]. Aptamers are also cheaper than antibodies, and can be recycled once attached to a solid surface of a column [22]. Aptamers can be developed in vitro selection [59,60], they are used in fast and on-site detection of food contaminants, and are recommended as an alternative in mycotoxin and toxigenic fungi detection [61,62]. The aptamers have been unable to be commercialized despite their advantage over others bioreceptors. They have significant disadvantages in terms of difficulty in the design of aptamers for small-sized molecules. Because of this problem, the improvement of binding affinity of aptamers with their targets may require a large number of complementary methods [63]. Monoclonal oligonucleotides (RNA or DNA) constitute the structure of aptamers, which have the ability to bind with high specificity to many targets [25]. Xia et al. [64] presented dual-terminal proximity aptamer probes for the detection of AFB1, an enzyme-free amplified and very fast method, with the whole process taking place in one tube. Detection of AFB1 was completed within 1 min, making this test one of the fastest tests for AFB1. When this structure was implemented, AFB1 was detected and, the recovery rates were 90.3% and 114.8% in bean paste and peanut oil samples, respectively.

## 3. Analytical Techniques in Analysis and Detection of Mycotoxins

The establishment of maximum permissible limits in many countries of the world requires the application of techniques capable of delivering accurate and reliable results in the mycotoxin analysis [65]. Chromatography is the predominant analytical technique that is used in food and feed mycotoxin analysis [19]. Through chromatographic separations, qualitative and quantitative determination are achieved, in a flexible way. Nevertheless, chromatographic techniques have disadvantages since they are techniques where pretreatment of the sample takes long detection time, and an experienced analyst, costly instrumentation, and a considerable time for processing results are often required [66]. The presence of mycotoxins at very low concentrations, the coexistence of many mycotoxins in the same food matrix, and their different chemical structures give rise to additional difficulties in their identification by analytical techniques [67].

Among the chromatographic techniques, TLC is considered the oldest and can be used for rapid screening of mycotoxins. It is a low-cost technique, but the measurements cannot be considered accurate and sensitive [68]. Moreover, the sample preparation step is necessary, and the physical and chemical structure of each mycotoxin determines the clean-up step [43]. Examples of TLC techniques for mycotoxin detection are presented in Table 2.

Gas chromatography is rarely used in the analysis of mycotoxins because the plethora of mycotoxins are nonvolatile and polar substances, and a derivatization step for their conversion in volatile derivatives is needed [19,65]. Usually this is accomplished by the silylation or acylation that occurs after clean-up [22]. Among electron capture (ECD), flame ionization (FID) and single mass spectrometry (MS) detectors, the latter is the most widely used in GC analysis. Analyzers, such as ion trap and quadrupole have been also utilized [65]. The analyzer time-of-flight (TOF) has been used for the analysis of TCs in wheat [75]. Moreover, the triple quadrupole (QqQ) in the GC–QqQ-MS/MS technique has been presented by Rodríguez-Carrasco et al. [76] for TCs, PAT, and ZEA analysis in wheat semolina. 

The choice of analytical technique is primarily related to the nature of the mycotoxin [66]. HPLC combines high resolution with more and more state-of-the-art automation [77]. Conventional detectors that are used in HPLC mycotoxin analysis are the fluorescence (FLD), UV-visible (UV), photodiode array (PDA), and MS (single mass spectrometry, and tandem MS (MS/MS)) [78]. HPLC with FLD or UV detectors can be used for the determination of chemically related mycotoxins [22]. HPLC coupled with FLD is the predominant technique for the quantification of OTA, with many advantages like good reliability and sensitivity in a single run [79], without the need for the presence of a chromophore, as it has natural fluorescence [78]. However, for other type of mycotoxins like FBs, derivatization is a mandatory step, as they don’t have chromophores in their chemical structure [80]. Many publications have reported the utility of HPLC–FLD for the analysis of AFs, ZEA, and DON [78]. HPLC coupled with PDA has been used for the detection of ATs [81], and an HPLC–PDA–FLD system was utilized for the simultaneous determination of AFs, DON, OTA and ZEA in wheat bran [53]. 

It is known that more than one mycotoxins can occur in various agricultural products. Consequently, this evidence has led the scientific community to discover new techniques capable of simultaneously identifying many mycotoxins [82]. Nowadays, MS/MS is used for accurate mass information [82] and the LC–tandem MS (MS/MS) technique is considered to be the most modern and widely used for mycotoxins analysis at trace levels, as it is more sensitive, specific and reliable compared to HPLC [49,83,84]. This technique has been successfully used for the simultaneous quantification of mycotoxins with different chemical structures [22] in one single run [34,36]. The European Committee for Standardization (CEN) has recently published the first official methodology for the ΖΕΝ analysis in edible vegetable oils, as well as T2 and HT2 mycotoxins in cereals and cereal products [85,86]. The LC/MS-MS technique has been reported by many studies in multimycotoxin determination, such as, 12 *Fusarium* mycotoxins in beer [87], 17 different mycotoxins in barley and malt [88] and 35 mycotoxins in medicine matrices [89]. Table 3 shows examples of LC/MS-MS methods in mycotoxin analysis in foods worldwide during 2014–2019. 

## 4. Rapid Diagnostic Methods for Mycotoxin Detection

### 4.1. Immunoassay-Based Methods 

Rapid diagnostic methods are mostly based on immunochemical assays, with major examples being ELISA, dipsticks, flow-through membranes and LFDs. Among all the immunological methods, ELISA is the most important tool for the rapid detection and quantification of mycotoxins [19]. The principle of the technique is the interaction of the antigen–antibody complex with the chromogenic substrates, and on the measurable result obtained by spectrophotometric measurement of the developed color [65]. ELISA methods have been developed for AFs, ZEA, OTA, DON, T2/HT2 and FBs testing in different agricultural commodities [98,99,100]. 

Dipstick has a similar principle of operation to that of ELISA, and flow-through membrane-based immunoassays provide qualitative and semiquantitative results. Because many of their results are approaching the cut-off level, all these immunoassays present poor commercial performance [19,24], although they are quick and give results that can be used within minutes. 

LFDs (also named immunostrips or immunodipsticks) are fast, in situ screening tools for immunechromatographic tests that work in a competitive way, having a labeled antibodies as a signal reagents [101] and working as pregnancy tests. The results from these tests are positive or negative, and come from visual evaluation. Portable photometric strip readers can also be used for obtaining results [65]. Signal amplification in LFDs has been achieved through novel materials, such as quantum dots (QDs) [102], gold nanoparticles (AuNPs) [103], magnetic nanoparticles (Fe_3_O_4_) [104], and carbon nanoparticles (CNPs) [105]. Although this method has great advantages, a major reason for limiting the use of LFDs is related with the interferences they cause. Moreover, it is a complicated matrix for the identification of trace analytes [22]. Their limited application is linked with reproducibility, reliability with different matrices, and sensitivity [24]. Some examples of LFDs for the detection and quantification of mycotoxins are listed in Table 4.

### 4.2. Biosensors in Mycotoxins Detection

The use of biosensors in the food industry can contribute to reducing the presence of mycotoxins by providing significant benefits such as fast, easy and inexpensive sample analysis, reproducibility, stability, accuracy, and on-site testing of samples [111,112]. Oliveira et al. recently presented advances in the use of biosensors in the detection of mycotoxins in food [25]. The transducers that are mainly used for mycotoxin detection are optical (surface plasmon resonance—SPR and fluorescence), piezoelectric (quartz crystal microbalance—QCM), and electrochemical (impedimetric, potentiometric and amperometric) [27,113]. Common recognition elements are peptides, enzymes, antibodies, cells, nucleic acids, but other materials such as aptamers, molecularly imprinted polymers and recombinant antibodies may also be used [25]. Metal nanoparticles, carbon nanotubes and nanofibers have been tested to improve the sensitivity of the biosensor. These materials are biocompatible and are characterized by special physicochemical characteristics, such as high surface-volume ratio [114,115]. The use of biosensors in mycotoxin detection with some examples are listed in Table 5.

#### 4.2.1. Electrochemical Biosensors for Mycotoxins Detection

##### Impedimetric Sensors 

The electrochemical impedance spectroscopy (EIS) technique has been developed to identify mycotoxins. This technique records the alterations observed in the interface between the electrode and the redox probe [138]. Three electrodes constitute an impedimetric sensor, the working, the reference and the counter electrode. Impedimetric sensors have been successfully tested for AFB1, AFM1, OTA and PAT [117,124,130,133]. 

##### Potentiometric Sensors 

The potentiometric sensors employ ion-selective electrodes. For this technique, two (working and reference) or three (working, reference and counter) electrode systems might be employed. The information on the recognition event is provided by the changes in circuit potential between the working and reference electrodes [139]. For the mycotoxin determination in foods, differential-pulse voltammetry (DPV), cyclic voltammetry (CV), and square-wave voltammetry (SWV) have been used [61]. Potentiometric sensors have been successfully tested for AFB1 in corn powder [120], for OTA in grape juice and red wine [129], for PAT in juice [132] and for ZEN in maize [136].

##### Amperometric Sensors 

For the technique of the amperometric sensor, two (working and reference) or three (working, reference and counter) electrode systems are required. Amperometric biosensors calculate currents that are produced through electroactive species. The input of mediators can improve the efficiency of the amperometric sensor by enhancing electron transfer [140,141]. An inert metal such as Pt or Au can be used as the working electrode, or alternatively, carbon nanotubes and graphene can also be used. 

The regeneration observed between measurements should be particularly careful, as it is a drawback of the technique. Nowadays, disposable printed electrodes are used instead, because they are cheap and can be produced in large scale [142,143]. Amperometric sensors have been successfully tested for HT-2 toxin, T-2 toxin, and AFM1, in human urine [113], for OTA in red wine [126] and for ZEN in corn and corn products [134,135].

#### 4.2.2. Optical Biosensors for Mycotoxins Detection

##### Surface Plasmon Resonance Sensors 

Optical biosensors show characteristics such as high specificity, sensitivity and cost-effectiveness. Optical biosensors also allow direct detection in real time. Important methods in the class of optical biosensors are surface plasmon resonance (SPR) and fluorescence resonance energy transfer (FRET). SPR is an uncomplicated, innovative analytical method, which gives fast results with high sensitivity. Moreover, through this technique, a label-free detection is performed as well as qualitative and quantitative analysis of multiplexed pollutants in real-time [144,145]. SPR-based biosensors have been tested for DON, ZEN and T-2toxin in wheat [121], for OTA in coffee [128], and for AFM1 in milk [130].

#### 4.2.3. Piezoelectric Biosensors for Mycotoxins Detection 

##### Quartz Crystal Microbalance (QCM) 

QCM-based biosensors have been investigated in both mycotoxin analysis and pathogen monitoring. The QCM transducer has a gold-plated crystal quartz, which by sending an electrical signal, modifies the resonant frequency. On the surface of quartz there is a sensory layer of interest through which mass change and specific vibrations are caused [146]. QCM-based biosensors have been tested for AFB1 in peanut, pistachio, rice, and wheat [116,119] and for OTA in red wine [127]. Advantages and limitations of each category of biosensors are presented in Table 6.

## 5. Emerging Technologies in Analysis and Detection of Mycotoxins

### 5.1. Proteomic and Genomic Methods 

Proteomic methods include initial mould peptide/protein extraction from food and further analysis by matrix-assisted laser desorption or ionization time-of-flight mass spectrometry (MALDI-TOF MS). The MALDI–TOF MS technique quickly detects fungal isolates and mycotoxins and identifies them with high precision, and is used alternatively in chromatographic techniques [28,150]. This analysis is performed by detecting proteins in a mass range of 2–20 kDa after calculating the m/z values. The identification of the micro-organism is achieved through the fingerprint MS created for each one. The MALDI-TOF MS technique is a very hopeful approach as it identifies closely related species of filamentous fungi. However, the need to create a public database in which all in-house entries are accessible has been reported. Additionally, the food industry currently considers this technique as expensive [151]. MALDI–TOF MS has been used to rapidly detect AFB1, CIT, DON, ZEA, T2, and griseofulvin [152], and for rapid screening of *Alternaria* mycotoxins, alternariol (AOH), alternariol monomethyl ether (AME) and tentoxin (TEN) [153].

Genomic methods include initial mould DNA or RNA extraction from foods followed by detection using polymerase chain reaction (PCR), quantitative PCR (qPCR), loop-mediated isothermal amplification (LAMP), and reverse transcription PCR (RT-PCR). Through these methods, fungal isolates are detected, but it is not possible to quantify them. It is also not possible to detect specific species, but only to detect strains with the ability to produce mycotoxins [28,154]. Through the PCR technique, amplification of DNA sequences in vitro is achieved. Selective and repetitive amplification requires the existence of the primers and Taq DNA polymerase. The real-time combination of the sensitivity of conventional PCR with a specific fluorescent signal allows qPCR to quantify specific DNA targets. LAMP uses a DNA polymerase and four primers in order to amplify nucleic acids, and it is used alternatively to PCR for the detection of spoilage moulds. OTA-producing fungi have been detected by PCR-based methods [155], *Fusarium*, *Penicillium* and *Aspergillus* species have also been detected by the same method, as well as analyzed as associated with the expression of genes involved in the biosynthetic pathway of several mycotoxins [28].

### 5.2. Molecular Techniques 

Among others, molecular techniques include PCR, fluorescent in situ hybridization (FISH) and DNA barcoding. Techniques that are varied with PCR such as qPCR, RT-PCR, PCR denaturing gradient gel electrophoresis (PCR–DGGE), and PCR–ELISA, can be used to control fungi in food [156]. Among them, PCR–DGGE has the advantage of terminal-restriction fragment length polymorphism analysis (T-RFLP), achieving satisfactory identifications for toxigenic fungi [29]. PCR–ELISA when compared to simple PCR, proved to be more sensitive. DNA amplification through LAMP was reported by Luo et al. [157] in order to detect aflatoxin-producing *Aspergillus* species.

### 5.3. Electronic Nose 

The physicochemical properties of secondary fungal metabolites can be assessed by the electronic nose in a way that works partially like a GC system. Specifically, this analysis is based on the detection of volatile compounds released by a contaminated food through a solid state sensor [158]. The sensitivity of the sensors that are used in this analysis allows the creation of a unique fingerprint for each food, characteristic of its taste and aroma. The detection of the characteristic odor gives initial information about the category of the produced metabolites [159,160]. Apples, oranges, strawberries and peaches are some fruits in which the application of this technique has been successfully implemented for the detection of fungi that produce mycotoxins [161,162,163,164].

### 5.4. Aggregation-Induced Emission Dye 

The aggregation-induced emission (AIE) exploits the enhancement of the fluorescence for a group of fluorescent dyes in the aggregation state. Intense fluorescence of these dyes may be the result of reduced intramolecular rotations observed in the aggregate state. The development of fluorescent biosensors is based on the fluorescence analysis of AIE dyes [165,166,167,168]. AIE dyes that show high fluorescence emission in the aggregate states, are 9,10-distyrylanthracene (DSA), tetraphenylethene (TPE), and silacyclopentadiene (silole) [166]. 

Zhu et al. [169] developed an AIE dye-based aptasensor for the detection of OΤA. AIE dyes showed high affinity to aptamers and fluoresce through the process of dye aggregation. Only one aptamer sequence was used in this study, with the detection limit reaching 0.4 ng/mL. In addition, it had significant specificity for the recognition of OTA. Moreover, the application was successfully used to analyze OTA in wine and coffee. The on-site detection of food contaminations and the simple operation make the application of AIE dyes very effective.

### 5.5. Quantitative NMR

Nuclear magnetic resonance (NMR) spectroscopy is a basic technique for the identification of organic compounds, and one of the significant analytical technologies frequently utilized in metabolomics [170]. Metabolomics is defined as the simultaneous detection and statistical interpretation of multiple endogenous metabolites in a living system. NMR-based metabolomics gives rich structural information by providing a ‘holistic approach’ of metabolites. The most commonly used NMR methodologies are based on studying the physical properties of hydrogen nuclei (protons) in tissue water, followed by proton NMR on other endogenous metabolites, and less frequently, other nuclei such as ^31^P, ^13^C, ^19^F and ^23^Na. Low sensitivity, low spectral resolution and poor time resolution are some of the disadvantages of NMR technology. Limited commercial software on the market and limited quantification methods also characterize this technique [171]. NMR has also several advantages such as straightforward sample preparation, high sample throughput, stable chemical shifts, quantification without standards, and reliable identification of isolate metabolites [172]. NMR technologies have been successfully used to elucidate rearrangement mechanism of the *Fusarium* mycotoxin Fusarin C [173]. 

### 5.6. Hyperspectral Imaging

Hyperspectral imaging (HSI) is a technique that can be applied for fungal and mycotoxin assessment with significant advantages. Analyses by this technique are inexpensive, are performed without destroying the sample, are quick, and operate by obtaining spectral data for each pixel location. This technique is considered to be capable of replacing expensive and time-consuming techniques for mycotoxins analysis in cereals, contributing significantly to the screening of contaminated cereal grains. The review by Femenias et al. [31] describes the principle of HSI technique and the use of HSI for *Fusarium* pathogen and DON risk management in cereals. Also, HSI prediction algorithms are important innovative data for DON determination. HSI is an important grain-sorting tool with the ability to classify individual contaminated grains. An HSI system consists of a camera for spectral and spatial detection, a spectrograph for the production of a spectrum for each pixel, an objective lens which focuses the beam of light in the direction of the detector, an illumination device that produces and sends light to the sample [174,175], a moving unit with a translation stage and motor which are responsible for the movement of the sample, and a data acquisition instrument [176].

Tekle et al. [177] studied a short wavelength infrared camera with a mercury telluride detector for the monitoring of *Fusarium* and DON contaminated cereal kernels. The working wavelengths of the device were 1000–2500 nm. The HSI presented by Barbedo et al. [178] was a XENICS camera coupled to a VIS/NIR spectrometer with working wavelengths of 528–1785 nm. They investigated the use of HSI for DON screening in wheat kernels through the use of a new algorithm. The results showed that the algorithm designed exclusively for this study did not provide sufficient evidence for the correlation between DON infection and the presence of *Fusarium*. A new algorithm was designed to categorize the samples into batches. The new algorithm that emerged could be a valuable tool for detecting initial batches of wheat followed by DON analysis. 

Recently, Ropelewska and Zapotoczny [179] developed images for the separation of healthy and infected cereals, using a charge coupled device camera, a VIS/NIR spectrometer with working wavelengths of 400–1100 nm and a fiber optic illuminator coupled with an infrared lamp. In a recent study, Liang et al. [30] used a charge coupled device camera and a spectrometer with working wavelengths of 400–1000 nm in order to identify and visualize the different DON levels in bulk wheat kernels. 

## 6. Conclusions 

As mycotoxins are responsible for food contamination and certain permissible limits have already been established, developing sensitive and reliable methods to detect them is a top priority. Before the detection and quantification of mycotoxins from contaminated samples, various extraction and clean-up protocols are applied. As the important points at the sample preparation techniques are the reduction of analysis time, small solvent volumes, and scale of extraction, extraction techniques with these advantages must be selected. Moreover, although a large number of analytical techniques are constantly being optimized and validated, and many novel methods continue to be developed, the LC/MS-MS technique is the fundamental tool for analyzing multiple mycotoxins. This technique, with its high sensitivity, accuracy and reliability, achieves the analysis of many mycotoxins in the same matrix. Factors that could limit the widespread use of chromatography include expensive equipment, specialized personnel, and complicated sample preparation protocols. Therefore, the use of chromatography for cases where fast and on-site analyzes are required, such as analyses by importers, traders and food and feed companies, is limited. Thus, if there is need for rapid mycotoxin determination in a “point-on-demand” format, immunoassay-based methods like the rapid immunoassays and biosensors must be used.

## Figures and Tables

**Table 1:** Extraction methods and solvents of mycotoxins.

Extraction Methods	Extraction Solvents	Limits	Benefits	Reference
QuEChERS	Organic solvents or mixtures (CH_3_CN, MeOH, MeOH/CH_3_CN)	Modifications of the original procedure, need of an additional enrichment step	Economical, fast, simple, detection of low ppb levels, better reproducibility and accuracy	[5]
LLE	Mixture of organic solvents (hexane, cyclohexane) with diluted acids or water	Time consuming, the sample can be absorbed by the glass equipment depending on the matrix and the determined compounds	Effective, for small-scale preparations	[40]
SLE	Mixture of organic solvents with diluted acids or water	Matrix effects	Smaller volumes of solvent	[32,45]
ASE or PLE	Mixture of organic solvents (MeOH/CH_3_CN, CH_3_CN/water)	Expensive instruments, matrix components excessively coextracted	Fully automated, faster extraction compared to the conventional ones, minimal solvents, higher extraction efficiency	[22,46]
SFE	Supercritical fluids (CO_2_), MeOH, ethanol, acetone	Need for specialized and very expensive equipment, not suitable for routine analysis	Fast technique, small solvent volumes, preconcentration effect, extraction of temperature sensible analytes	[22,45,47]
MAE	Aqueous solution	Only applicable for thermally stable compounds, costly instruments	Reduced extraction time and extraction solvent	[48]
VALDS–ME	Mixture of organic solvents dispersive solvent and water	Optimization after control a lot of parameters	Use of low density solvents, simple, fast, effective	[20]

**Table 2:** Examples of thin-layer chromatography (TLC) techniques for mycotoxin detection.

Sample	Origin	Number of Samples	Mycotoxins	LOD	LOQ	References
Herbs and herbal products	India	63	AFB1, AFB2, AFG1, AFG2, CIT	10 ng/mL for AFB1 NA for others	NA	[69]
Herbal medicines	Nigeria	210	AFB1, AFB2, AFG1, AFG2	NA	NA	[70]
Brazil nuts	Brazil	67	AFB1, AFB2, AFG1, AFG2	NA	2 mg/kg	[71]
Almonds, cashew nuts, chestnuts, hazelnuts, pistachio nuts, walnuts	Saudi Arabia	5	AFB1, AFB2, AFG1, AFG2, CIT, OTs, PAT, T-2, ZEA, ST, DAS	NA	5 μg/kg (for AFs), NA for others	[72]
Medicinal plants	Pakistan	30	AFB1, AFB2, AFG1, AFG2, OTA	NA	NA	[73]
Corn-based food products	Brazil	208	AFB1, AFB2, AFG1, AFG2	NA	2 µg/kg	[74]

NA-not available in the publication. ST sterigmatocystin, DAS diacetoxyscirpenol.

**Table 3:** Examples of liquid chromatography with tandem mass spectrometry (LC/MS-MS) methods in mycotoxins analysis in foods worldwide during 2014–2019.

Mycotoxin	Year of Publication	Country	Sample	Extraction Solution	Extraction Method	Clean-Up	LOD	LOQ *	Reference
AFs	2014	China	Walnut kernel	Methanol–water (70:30, *v*/*v*)	Sonicating	Self-made amino-function nanometer Fe3O4 magnetic polymer SPE	0.004–0.013 µg kg−1	0.012–0.042 µg kg−1	[90]
AFs, OTA *Fusarium* mycotoxins	2014	Italy	Cereals and derived products	Methanol–water (60:40, *v*/*v*)	Blending	IAC	1 µg kg−1 for AFs and OTA 5–30 µg kg−1 for *Fusarium* toxins	Nd	[52]
5 *Alternaria* mycotoxins, CIT	2015	Belgium	Tomato and tomato juice	Methanol 2,4-dinitrophenylhydrazine	Vortex	SPE cartridge	1–20 µg kg^−1^	2–50 µg kg−1	[91]
4 *Alternaria* mycotoxins	2016	China	Wheat kernel	Acetonitrile–water–methanol (45:45:10, *v*/*v*/*v*)	Sonicating	SPE cartridge	0.04–1.3 µg kg^−1^	0.1–4.2	[92]
AFs, FB1, FB2, DON, OTA, ZEA	2016	Thailand	Brown rice	Acetonitrile with 10% (*v*/*v*) acetic acid	Vortex	QuEChERS	1.4–25 µg kg^−1^	4.1–75 µg kg−1	[93]
15 mycotoxins	2016	Spain	Cow milk	Acetonitrile (2% formic acid)	Shaking	Sodium acetate	0.02−10.14 ng mL^−1^	Nd	[94]
16 mycotoxins	2017	China	Vegetable oils	85% Acetonitrile	Shaking	QuEChERS	0.04–2.9 ng g^−1^	Nd	[95]
11 mycotoxins	2017	USA	Infant cereals	Acetonitrile/water/formic acid, (80:19.9:0.1, *v*/*v*/*v*)	Shaking	Nd	0.01−10.0 ng g^−1^	0.05–50 ng g–1	[84]
12 *Fusarium* mycotoxins	2017	Germany	Beer	Acetonitrile/water (70:30, *v*/*v*) Acetonitrile/water (84:16, *v*/*v*)	Vortex	SPE cartridge	0.05–6.9 µg L^−1^	0.15–20 µg L−1	[87]
AFB1 OTA FB1 DON T2 HT-2 ZEA	2017	Italy	Cereal-based samples	Acetonitrile–water–acetic acid (79:20:1, *v*/*v*/*v*)	Shaking	Nd	0.06–0.13 µg kg^−1^fo r AFB1 0.4–0.8 µg kg^−1^ for OTA 8−16 µg kg^−1^ for FB1 20 µg kg^−1^ for DON 4–8 µg kg^−1^ for T–2 20 µg kg^−1^ for HT–2 1.6–3.2 µg kg^−1^ for ZEA	Nd	[96]
13 mycotoxins	2017	Korea	Cereal grains	Methanol 80%, containing 0.5% acetic acid	Shaking	IAC	0.1−18.1 ng/g	0.4–54.8 ng/g	[97]
20 mycotoxins	2019	Korea	Soybean Paste	Methanol–water (60:40, *v*/*v*) and PBS	Blending	IAC	0.06–4.68 µg kg^−1^	0.17−13.24	[49]
6 *Alternaria* toxins	2019	China	Grapes	Acetonitrile and dispersive solid phase extraction	Shaking	QuEChERS	0.03–0.21 µg kg^−1^	0.09–0.48 µg kg−1	[77]
AFs, ZEA, α-ZOL	2019	Spain	Vegetable oils	Acetonitrile	Shaking	QuEChERS	Nd	0.5 μg kg−1 for AFs 1 μg kg−1 for ZEA and α-ZOL	[93]

α-ZOL, α-zearalenol; CIT, citrinin * Nd (not described).

**Table 4:** Some examples of lateral-flow devices for detection and quantification of mycotoxins.

Mycotoxin	Label Used	Commodity	Sensitivity	Reference
Deoxynivalenol (DON) Zearalenone (ZEA) T-2/H-T2-toxin	Epoxy-functionalized silica coated QDs	Barley	1000 µg/kg 80 µg/kg 80 µg/kg	[106]
Aflatoxin B1 (AFB1) Zearalenone (ZEA) Deoxynivalenol (DON)	Monoclonal antibodies (mAbs) with the conjugates bovine serum albumin (BSA)	Wheat and maize	0.05 μg/kg 1 μg/kg 3 μg/kg	[107]
Fumonisin B1 (FB1) Deoxynivalenol (DON)	Gold nanoparticles (AuNPs)	Maize	2.0 ng mL^−1^ 40 ng mL^−1^	[108]
Deoxynivalenol (DON) T-2 toxin (T-2) Zearalenone (ZEN)	Amorphous carbon nanoparticles (ACNPs)	Maize	20 µg/kg 13 µg/kg 1 µg/kg	[109]
Aflatoxin B1 (AFB1) Zearalenone (ZEN) Deoxynivalenol (DON)	CdSe/SiO2 quantum dot microbeads	Feedstuff	10 pg mL^−1^ 80 pg mL^−1^ 500 pg mL^−1^	[110]
Zearalenone (ZEN)	Antibody-labeled quantum dot sumicro beads	Corn	3.6 mg mL^−1^	[111]
Fumonisins (FUs)	CdSe/ZnS QD + GNP	Maize	62.5 μg/kg	[102]

**Table 5:** Use of biosensors in mycotoxin detection with some examples.

Mycotoxin	Recognition Element	Transducer/Technique	Food	Detection Limit	Reference
AFB1	Organic framework composite	Piezoelectric (QCM)	Peanut, pistachio, rice, and wheat	2.8 pg mL^−1^	[116]
AFB1	Antibody	Impedimetric (EIS)	Corn	0.05 ng mL^−1^	[117]
AFB1	Antibody	Piezoelectric (EQCM)	Cereal	8 pg mL^−1^	[118]
AFB1	Antibody	Piezoelectric (QCM)	Peanut	0.83 ng kg^−1^	[119]
AFB1	Antibody	Potentiometric (DPV)	Corn powder	3.5 pg mL^−1^	[120]
Cyclopiazonic acid	Antibody	Optical (SPR)	Maize and cheese	0.29 mg mL^−1^	[121]
DON, ZEN, T-2toxin	Antibody	Optical (SPR)	Wheat	15μg/kg^−1^ 24 μg/kg^−1^ 12 μg/kg^−1^	[122]
HT-2 toxin, T-2 toxin, AFM1	Antibody	Amperometric (CV)	Human urine	0.4 ng mL^−1^ 1 ng mL^−1^ 0.3 ng mL^−1^	[113]
T-2 toxin, T-2 toxin-3-glucoside (T2-G)	Antibody	Optical (iSPR)	Wheat	1.2 ng mL^−1^	[123]
OTA	Aptamer	Impedimetric (EIS)	Grape and commodities	0.030 ng mL^−1^	[124]
OTA	Aptamer	SPR	Wine and peanut oil	0.005 ng mL^−1^	[125]
OTA	Antibody	Piezoelectric (QCM)	Buffer	17.2 ng mL^−1^	[111]
OTA	Aptamer	Amperometric (CV)	Red wine	0.23 pg mL^−1^	[126]
OTA	Antibody	Piezoelectric (QCM)	Red wine	0.16 ng mL^−1^	[127]
OTA	Antibody	Optical (SPR)	Coffee	3.8 ng mL^−1^	[128]
OTA	Black phosphorene	Potentiometric (DPV)	Grape juice and red wine	0.18 μg mL^−1^	[129]
OTA	Antibody	Piezoelectric (QCM-D)	Red wine	0.16 ng mL^−1^	[127]
OTA, AFM1	Antibody	Potentiometric (CV)	Red wine and milk	0.15 ng mL^−1^ 3.04 ng mL^−1^	[130]
AFM1	Antibody	Optical (SPR)	Milk	18 pg mL^−1^	[131]
PAT	Aptamer	Potentiometric (EIS/DPV)	Juice	0.27 pg mL^−1^	[132]
PAT	Aptamer	Impedimetric (EIS)	Apple juice	2.8 ng L^−1^	[133]
ZEN	Aptamer	Amperometric (CV/DPV)	Maize	0.17 pg mL^−1^	[134]
ZEN	Antibody	Amperometric (CV/DPV)	Corn and corn products	1.5 pg mL^−1^	[135]
ZEN	Aptamer	Potentiometric (CV/DPV)	Maize	0.105 pg mL^−1^	[136]
DON, T-2, ZEA, FB1	Antibody	Optical (iSPR)	Barley	64 µg kg^−1^, 26 µg kg^−1^, 96 µg kg^−1^, 13 µg kg^−1^	[137]

**Table 6:** Advantages and limitations of biosensors in mycotoxin detection.

Biosensors	Advantages	Limitations	Reference
Impedimetric	High sensitivity and selectivity, time-efficient, simple operation, fast response, mobility due to portable instrumentation, miniaturization	Complex construction, expensive labeling markers	[25,124,147]
Potentiometric	Reduced analysis time, mobility due to portable instrumentation, miniaturization, high sensitivity and selectivity, use without sample treatment	The sensitivity and lifetime are seriously influenced by factors such as temperature, pH, immobilization support, and immunological cross-reaction	[129,147,148]
Amperometric	Mobility due to portable instrumentation, miniaturization, high sensitivity and selectivity	Regeneration between measurements	[147]
Surface plasmon resonance	High specificity and sensitivity, small size and cost-efficiency, direct, real-time analysis and detection without label, development of portable devices	The broad practical application is still under development	[25,144]
Quartz crystal microbalance	Low cost with high sensitivity, selectivity, and possibility of reuse, real-time output, and label- or radiation-free entities, development of portable devices	Requirement of a relatively high background signal relative to the signal-on assay formation	[119,149]
